# Screening key genes for abdominal aortic aneurysm based on gene expression omnibus dataset

**DOI:** 10.1186/s12872-018-0766-8

**Published:** 2018-02-13

**Authors:** Li Wan, Jingyong Huang, Haizhen Ni, Guanfeng Yu

**Affiliations:** 1 Department of pathology, The First Affiliated Hospital of Wenzhou Medical University, Wenzhou, Zhejiang, China; 2 Department of vascular surgery, The First Affiliated Hospital of Wenzhou Medical University, NO.3, YuanXi Lane, Lucheng District, Wenzhou, Zhejiang 325000 China

**Keywords:** abdominal aortic aneurysm, gene expression, protein-protein interaction network, TFs regulatory network, biomarkers, therapy target

## Abstract

**Background:**

Abdominal aortic aneurysm (AAA) is a common cardiovascular system disease with high mortality. The aim of this study was to identify potential genes for diagnosis and therapy in AAA.

**Methods:**

We searched and downloaded mRNA expression data from the Gene Expression Omnibus (GEO) database to identify differentially expressed genes (DEGs) from AAA and normal individuals. Then, Gene Ontology and Kyoto Encyclopedia of Genes and Genomes pathway analysis, transcriptional factors (TFs) network and protein-protein interaction (PPI) network were used to explore the function of genes. Additionally, immunohistochemical (IHC) staining was used to validate the expression of identified genes. Finally, the diagnostic value of identified genes was accessed by receiver operating characteristic (ROC) analysis in GEO database.

**Results:**

A total of 1199 DEGs (188 up-regulated and 1011 down-regulated) were identified between AAA and normal individual. KEGG pathway analysis displayed that vascular smooth muscle contraction and pathways in cancer were significantly enriched signal pathway. The top 10 up-regulated and top 10 down-regulated DEGs were used to construct TFs and PPI networks. Some genes with high degrees such as NELL2, CCR7, MGAM, HBB, CSNK2A2, ZBTB16 and FOXO1 were identified to be related to AAA. The consequences of IHC staining showed that CCR7 and PDGFA were up-regulated in tissue samples of AAA. ROC analysis showed that NELL2, CCR7, MGAM, HBB, CSNK2A2, ZBTB16, FOXO1 and PDGFA had the potential diagnostic value for AAA.

**Conclusions:**

The identified genes including NELL2, CCR7, MGAM, HBB, CSNK2A2, ZBTB16, FOXO1 and PDGFA might be involved in the pathology of AAA.

## Background

Abdominal aortic aneurysm (AAA), defined as the aortic diameter > 3.0 cm, is a cardiovascular system disease that is characterized by aortic dilation that exceeds the normal aortic diameter by more than 50%. AAA dilatation will lead to rupture of the aorta, which results in bleeding. Generally, it is asymptomatic until the rupture event occurs [1]. Additionally, AAA is common in adult patients, especially elderly men, and leads to severe complications [2–4]. Up to now, the etiology of AAA remains unclear. It is noted that some clinical risk factors including smoking history, advanced age, family history, hypertension, hyperlipidaemia, atherosclerosis, chronic obstructive pulmonary disease are remarkably related to AAA [3, 5–7]. It is also observed that the intricate interplay of apoptosis, inflammation and matrix degradation is involved in the development of this disorder [8–10]. Anyway, the pathophysiology of AAA is complex, but fundamentally aneurysm comes from the vessel wall structural integrity loss and the vessel wall weakening. It is pointed out that vascular smooth muscle cells are the critical cell type involved in the development of AAA [11].

In a word, AAA is a common and late onset disease, which can rupture with a high mortality if not treated. In some clinical practice, there is no effective treatment other than surgical approaches to repair AAA [12]. And endovascular aneurysm repair has improved detection and lower mortality rates of AAA [13–17]. However, morbidity and mortality after surgery are still common [18, 19]. Therefore, understanding the genetic architecture and pathological mechanism of the disease may provide valuable information for elucidation of pathogenic mechanisms and signal pathways in AAA and the discovery of potential biomarkers and drug targets in AAA diagnosis and non-surgical treatment therapy.

In this study, we tried to find differentially expressed genes (DEGs) in AAA by integrated analysis. Then, functional enrichment analysis including Gene Ontology (GO) and Kyoto Encyclopedia of Genes Genomes (KEGG) was used to investigate the biological function of DEGs followed by transcriptional factors (TFs) network an d protein-protein interaction (PPI) network construction of top 20 DEGs (10 up-regulated and 10 down-regulated). Immunohistochemical (IHC) staining was applied to validate the expression of candidate DEGs. Finally, receiver operating characteristic (ROC) analyses was applied to analyze diagnosis ability of identified DEGs. Our study may be helpful in understanding the pathogenic mechanism and finding valuable diagnosis biomarkers and therapy drug in AAA.

## Methods

### Datasets

In this study, we searched datasets from the Gene Expression Omnibus (GEO) database (http://www.ncbi.nlm.nih.gov/geo/) with the keywords abdominal aortic aneurysm [All Fields] AND (“gse”[Filter] AND “Homo sapiens”[Organism]). The study type was described as “expression profiling by array.” All selected datasets were genome-wide expression data of AAA group and/or normal group tissue samples. Those standardized or primary datasets were included in this study. Finally, a total of 3 datasets including GSE7084, GSE47472 and GSE57691 were screened, which was shown in Table 1.

**Table 1:** Three datasets in GEO

GEO accession	Author	Platform	Samples(P:N)	Year
GSE7084	Tromp G	GPL570[HG-U133_Plus_2] Affymetrix Human Genome U133 Plus 2.0 Array;GPL2507Sentrix Human-6 Expression BeadChip	7:8	2007
GSE47472	Biros E	GPL10558Illumina HumanHT-12 V4.0 expression beadchip	14:8	2013
GSE57691	Biros E	GPL10558Illumina HumanHT-12 V4.0 expression beadchip	49:10	2015

*P* patients, *N* normal individual

### Analysis of DEGs

Raw expression data of AAA patients in this study were downloaded. Limma and metaMA packages were used to identify the DEGs. And the inverse normal method was used to combine the *p* value in metaMA. The false discovery rate (FDR) was performed for multiple testing corrections of raw p value through the Benjamin and Hochberg method [20, 21]. The threshold of DEGs was set as FDR < 0.01.

### Functional annotation analyses of DEGs

To obtain the biological function and signaling pathways of DEGs, the Metascape software was used for Gene Ontology (GO, http://www.geneontology.org/) annotation and Kyoto Encyclopedia of Genes Genomes (KEGG, http://www.genome. jp/kegg/pathway.html) pathway enrichment of DEGs. The threshold of GO function and KEGG pathway of DEGs was all set as FDR < 0.05.

### PPI network construction

It is useful for understanding the molecule mechanism of AAA to study the interactions between proteins. In order to gain insights into the interaction between proteins encoded by DEGs and other proteins, the database of BioGRID (http://thebiogrid.org) was used to retrieve the predicted interactions between top 20 proteins encoded by DEGs (10 up-regulated and 10 down-regulated) and other proteins. The PPI network was generated by the Cytoscape Software (http://cytoscape.org/). A node in the PPI network denotes protein, and the edge denotes the interactions.

### Analysis of potential TFs to target DEGs

TFs play a critical role in regulating gene expression. We downloaded the TFs in the human genome and the motifs of genomic binding sites from the TRANSFAC. Moreover, the 2 KB sequence in the upstream promoter region of DEGs was downloaded from UCSC (http://www.genome.ucsc.edu/cgi-bin/hgTables). Target sites of potential TFs were then distinguished. Finally, the transcriptional regulatory network was visualized by Cytoscape software.

### Immunohistochemical (IHC) staining for CCR7 and PDGFA

In this study, a patient with AAA and a normal individual was enrolled for the IHC experiment. The 5 μm slides were incubated with anti CCR7 primary rabbit anti-human polyclone antibody (1:500 dilution; abcam) and anti PDGFA primary rabbit anti-human polyclone antibody (1:500 dilution; Invitrogen) followed incubated with peroxidase conjugated goat anti-rabbit secondary antibody (1:200 dilution; Vector). For color visualization, diaminobenzidine (DAB) substrate (Vector) was applied. The staining area was analyzed by the software of Image Pro-plus 6.0 (Media Cybernetics Corporation, arrendale, PA, USA), and quantified by the IHC staining score (intensity score × positive rate score). The negative (−), positive (+), positive (++), positive (+++) of intensity scores represented 0, 1, 2 and 3, respectively. The positive rate score including negative, 1–25%, 26–50%, 51–75% and 76–100% represented 0, 1, 2, 3 and 4, respectively. IHC staining score of 0, 1~ 4, 5~ 8 and 9~ 12 represented negative, slight positive, moderate positive and strong positive, respectively.

All patients provided written informed consent with the approval of the ethics committee of the First Affiliated Hospital of Wenzhou Medical University (2017147).

### Receiver operating characteristic analyses

By using pROC package in R language we performed the receiver operating characteristic (ROC) analyses to assess the diagnostic value of DEGs (NELL2, CCR7, MGAM, HBB, CSNK2A2, ZBTB16, FOXO1 and PDGFA) in AAA. The area under the curve (AUC) was calculated and the ROC curve was generated.

## Results

### DEGs analysis

**Fig. 1:**
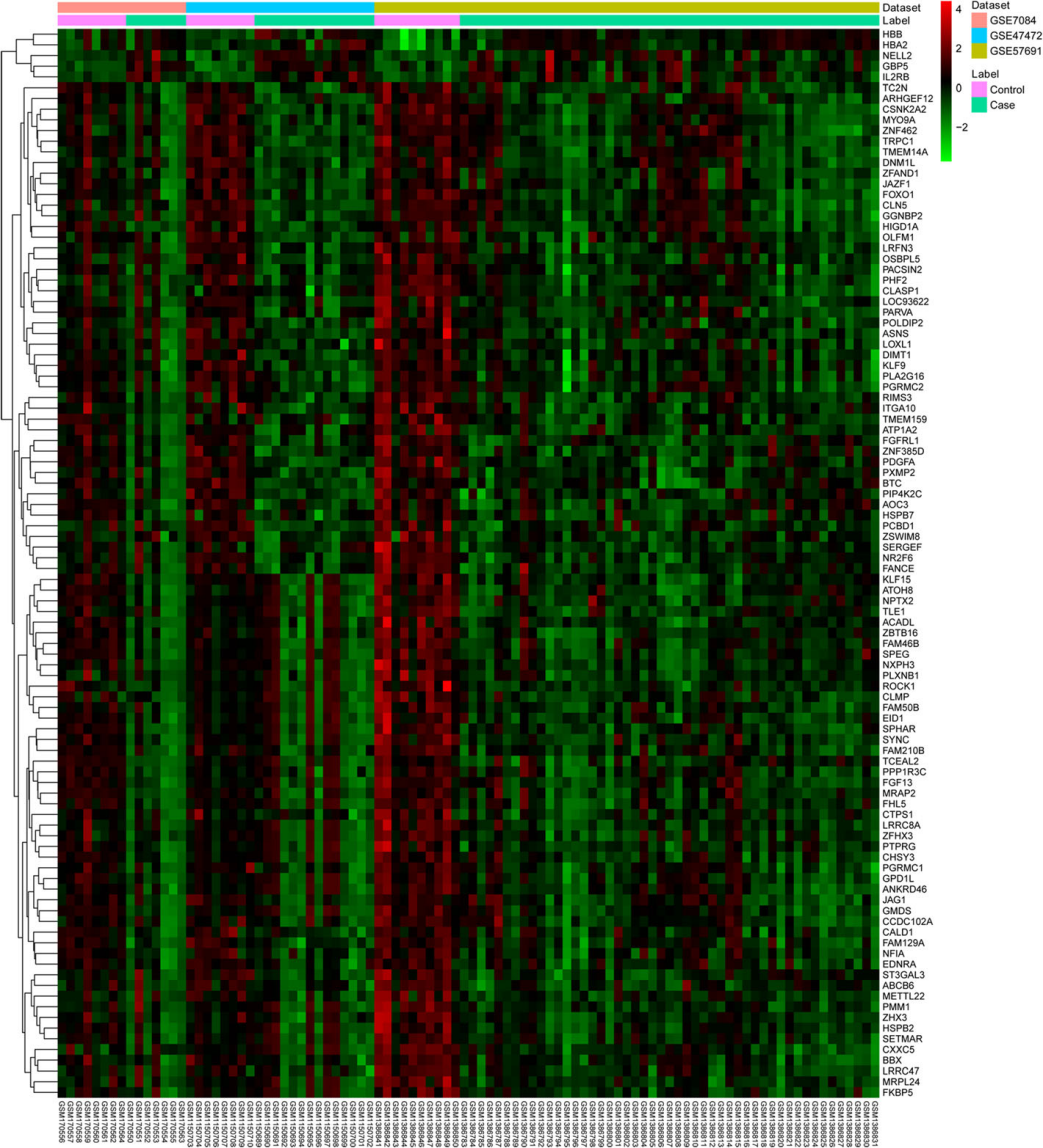
The heat map of top 50 DEGs. The diagram presents the result of a two-way hierarchical clustering of the top 50 DEGs and samples. The clustering is constructed using the complete-linkage method together with the Euclidean distance. Each row represents a DEG and each column, a sample. The DEG clustering tree is shown on the right. The colour scale illustrates the relative level of DEG expression: red, below the reference channel; green, higher than the reference

Raw expression profiles of AAA patients were downloaded from the data portal of the GEO database. A total of 1199 DEGs were identified as the threshold at FDR < 0.01, consisting of 188 up-regulated genes and 1011 down-regulated genes. The top 10 up- and down-regulated DEGs are listed in Table 2. The heat map of the top 50 DEGs is shown in Fig. 1.

**Table 2:** Top 10 up- and down-regulated DEGs

Gene ID	Gene Symbol	FDR	Combined.ES	Gene ID	Gene Symbol	FDR	Combined.ES
Up-regulated genes				Down-regulated genes			
115,362	GBP5	2.46E-07	1.718728703	5154	PDGFA	2.01E-10	−2.16796797
3043	HBB	5.91E-07	1.779962188	2063	NR2F6	3.18E-09	−2.07109705
3040	HBA2	1.45E-06	1.734233872	1459	CSNK2A2	3.18E-09	−2.009947802
3560	IL2RB	4.69E-06	1.434746719	28,999	KLF15	6.12E-09	−2.026442955
4753	NELL2	8.52E-06	1.395326	7220	TRPC1	7.99E-09	−1.919770839
5743	PTGS2	1.01E-05	1.378162107	81,493	SYNC	2.20E-08	−1.980299756
84,658	ADGRE3	1.03E-05	1.423979214	7704	ZBTB16	2.31E-08	−2.023343723
8972	MGAM	1.37E-05	1.372006422	116,151	FAM210B	4.86E-08	−1.869732488
54,504	CPVL	1.49E-05	1.4454994	2308	FOXO1	4.94E-08	−1.841528166
1236	CCR7	1.66E-05	1.335317111	58,499	ZNF462	5.47E-08	−2.004150438

*FDR* false discovery rate, *Combined.ES* combined effect size

### Functional and pathway enrichment analyses of DEGs

**Fig. 2:**
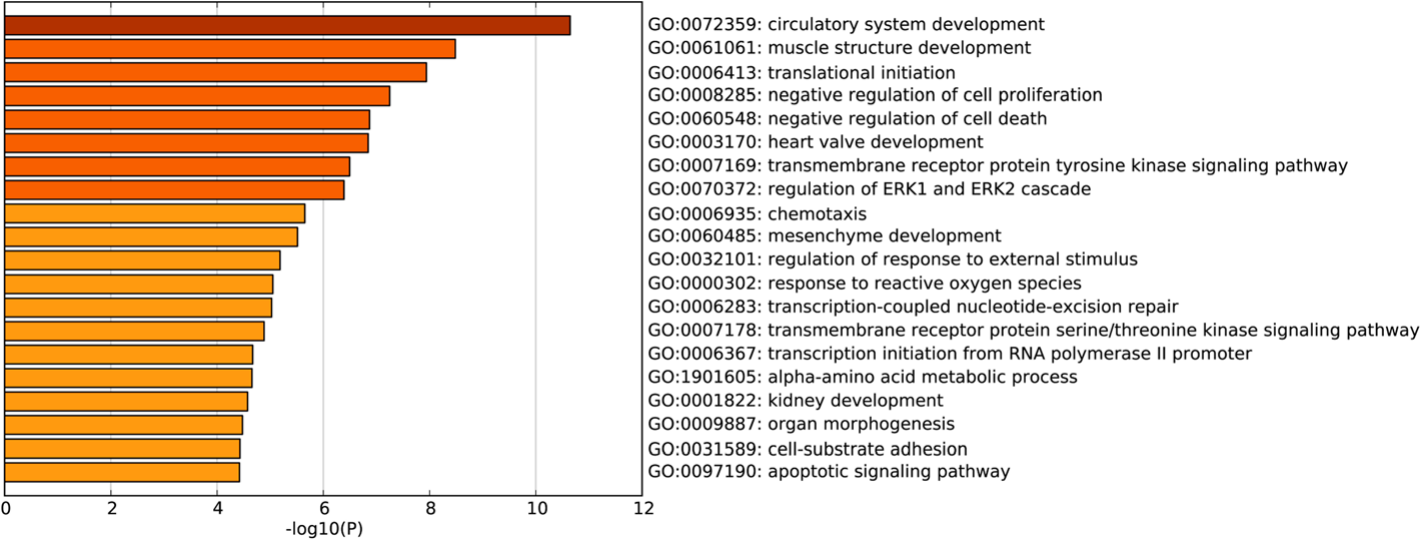
Significantly enriched biological processes of DEGs. The x-coordinate axis presents the FDR value. FDR value is more highly, the colour of the bar is more deeply

**Fig. 3:**
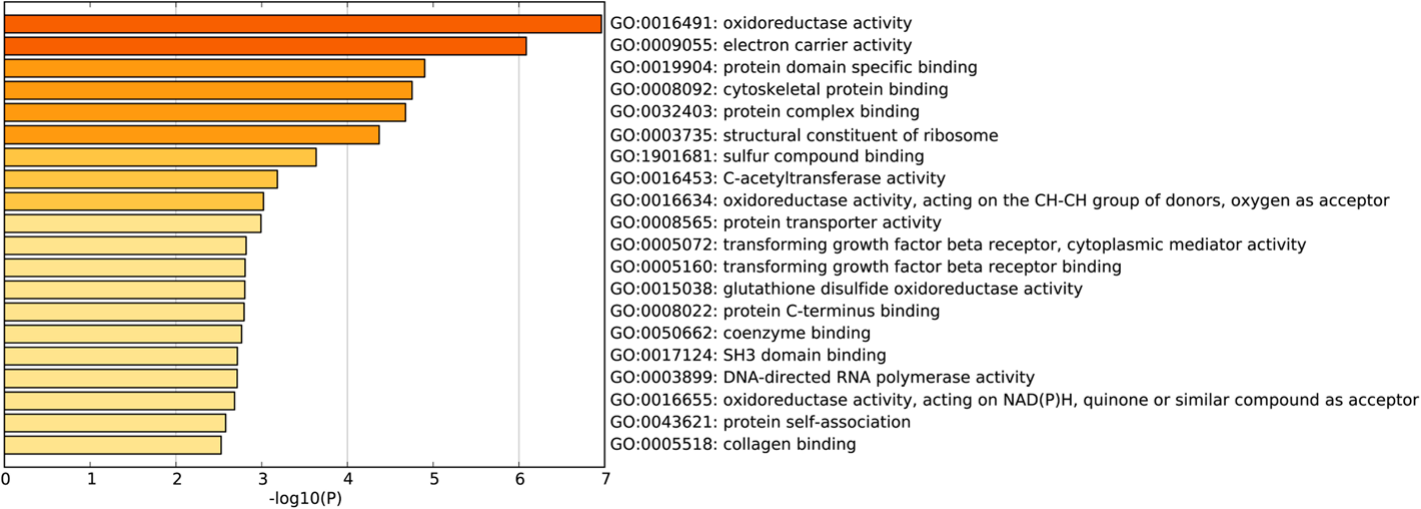
Significantly enriched molecular functions of DEGs. The x-coordinate axis presents the FDR value. FDR value is more highly, the colour of the bar is more deeply

**Fig. 4:**
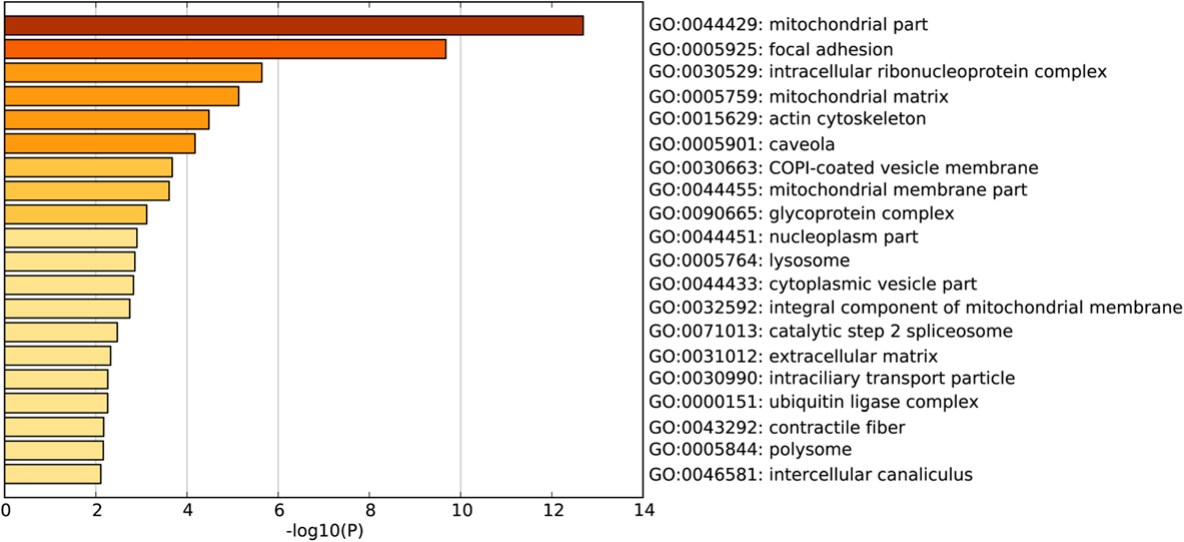
Significantly enriched cellular components of DEGs. The x-coordinate axis presents the FDR value. FDR value is more highly, the colour of the bar is more deeply

**Fig. 5:**
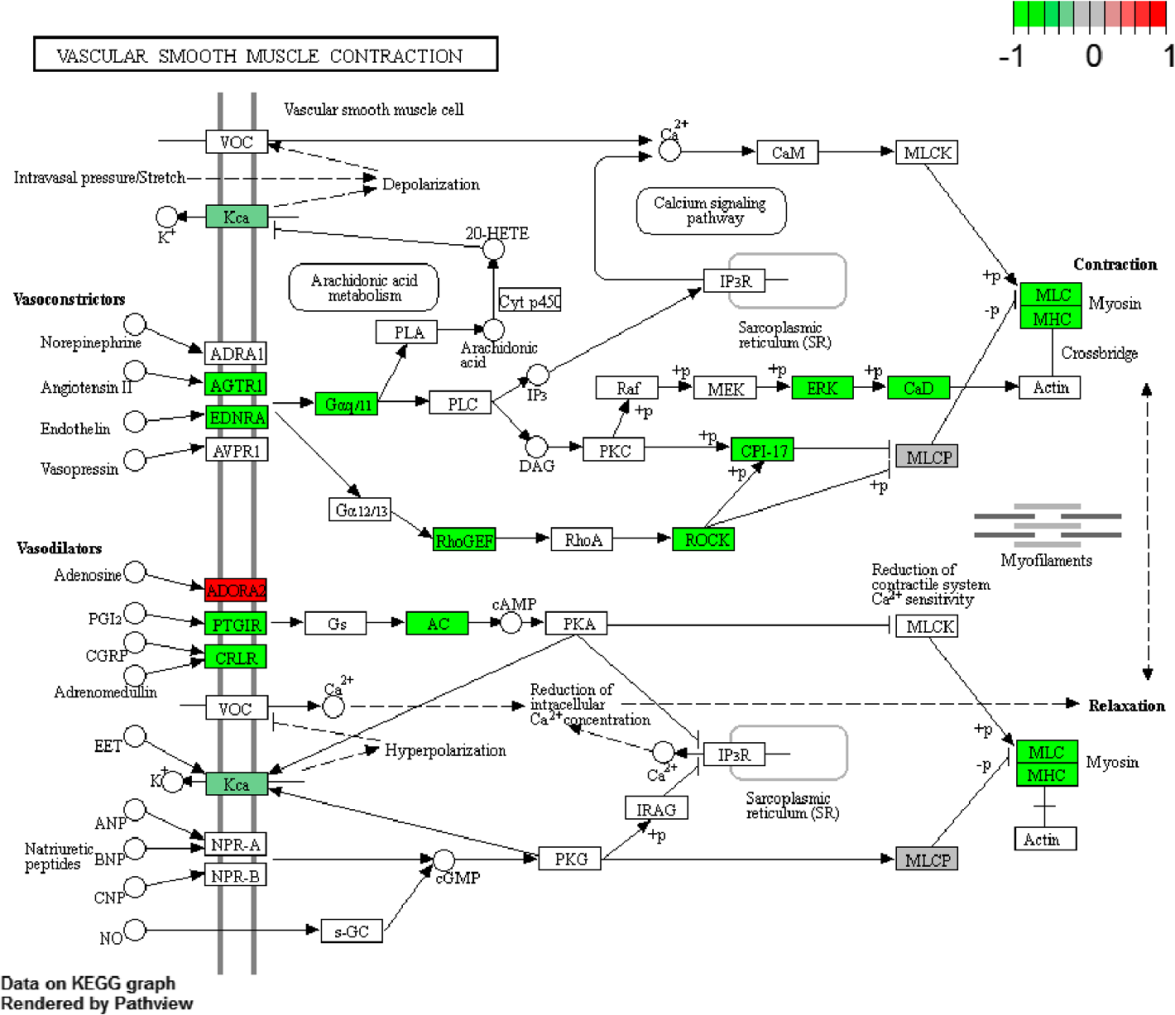
Significantly enriched vascular smooth muscle contraction signal pathways of DEGs. The red and green diamond represents the up and down-regulated DEGs

**Fig. 6:**
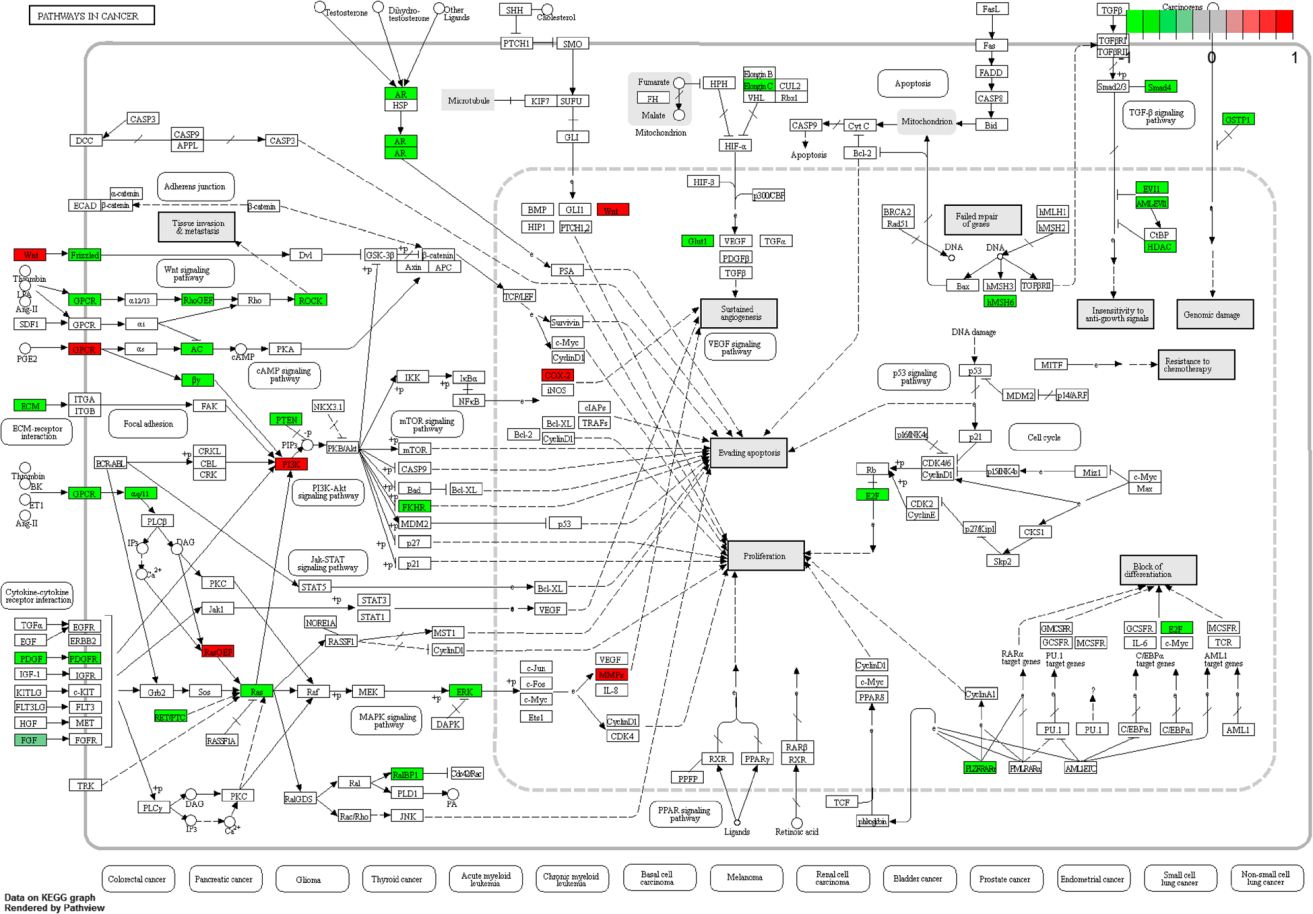
Significantly enriched pathways in cancer signal pathways of DEGs. The red and green diamond represents the up and down-regulated DEGs

To investigate the biological function of the identified DEGs in AAA, GO term and KEGG pathway enrichment analyses was performed. In GO term and KEGG pathway enrichment analyses, circulatory system development, muscle structure development and translational initiation were the most significant enrichment in biological process (Fig. 2); Oxidoreductase activity, electron carrier activity, protein domain specific binding were the most notable enrichment in molecular function (Fig. 3); Mitochondrial part, focal adhesion and intracellular ribonucleoprotein complex were the most significant enrichment in cellular component (Fig. 4). The top 10 GO terms of DEGs are shown in Table 3, and the KEGG enrichment signal pathways of DEGs shown in Table 4. The vascular smooth muscle contraction and pathways in cancer that were significantly related to AAA are shown in Fig. 5 and Fig. 6, respectively.

**Table 3:** Top 10 GO terms of DEGs

GO ID	GO term	List in term	Log p
Biological process			
GO:0072359	circulatory system development	101/913	−10.6462
GO:0061061	muscle structure development	67/563	−8.4846
GO:0006413	translational initiation	40/268	−7.9403
GO:0008285	negative regulation of cell proliferation	70/643	−7.2506
GO:0060548	negative regulation of cell death	89/910	−6.8694
GO:0003170	heart valve development	12/34	−6.8435
GO:0007169	transmembrane receptor protein tyrosine kinase signaling pathway	89/928	−6.4948
GO:0070372	regulation of ERK1 and ERK2 cascade	34/238	−6.3893
GO:0006935	chemotaxis	81/860	−5.6514
GO:0060485	mesenchyme development	32/237	−5.5128
Molecular function			
GO:0016491	oxidoreductase activity	75/719	−6.9626
GO:0009055	electron carrier activity	21/112	−6.0854
GO:0019904	protein domain specific binding	61/623	−4.9011
GO:0008092	cytoskeletal protein binding	74/810	−4.7540
GO:0032403	protein complex binding	82/928	−4.6775
GO:0003735	structural constituent of ribosome	27/210	−4.3707
GO:1,901,681	sulfur compound binding	27/232	−3.6356
GO:0016453	C-acetyltransferase activity	3/4	−3.1832
GO:0016634	oxidoreductase activity, acting on the CH-CH group of donors, oxygen as acceptor	4/9	−3.0208
GO:0008565	protein transporter activity	14/98	−2.9919
GO:0016491	oxidoreductase activity	75/719	−6.9626
Cellular component			
GO:0044429	mitochondrial part	109/943	−12.6878
GO:0005925	focal adhesion	55/391	−9.6768
GO:0030529	intracellular ribonucleoprotein complex	70/710	−5.6423
GO:0005759	mitochondrial matrix	45/404	−5.1349
GO:0015629	actin cytoskeleton	46/442	−4.4798
GO:0005901	caveola	14/76	−4.1752
GO:0030663	COPI-coated vesicle membrane	6/17	−3.6750
GO:0044455	mitochondrial membrane part	22/173	−3.6079
GO:0090665	glycoprotein complex	6/21	−3.1162
GO:0044451	nucleoplasm part	59/708	−2.9012

List in term: the number of DEGs on the total number of genes in GO term

*Log p* logarithm processing of *p* value

**Table 4:** The KEGG enrichment signal pathways of DEGs

KEGG ID	KEGG term	List in term	Log p	Gene list
hsa03010	Ribosome	22/135	−6.5000	FAU,RPL7,RPL9,RPL24,RPL27,RPL30,RPL35A,RPS6,RPS21,UBA52,MRPL33,MRPL19,MRPL18,MRPL22,MRPS16,RSL24D1,MRPL20,MRPS15,MRPS6,MRPS5,MRPL1,MRPL24
hsa00640	Propanoate metabolism	10/32	−5.9097	ACAT1,ACAT2,LDHA,LDHB,ALDH6A1,MUT,PCCA,SUCLG2,HIBCH,ACSS2,ALDH2,ALDH3A2,HADH,HMGCL,ACO1,GCSH,HOGA1,ESD,PFKM,PRPS2,PHGDH,L2HGDH
hsa04510	Focal adhesion	23/202	−4.1028	ACTN1,CAPN2,CAV2,COL4A1,FLNC,HRAS,ITGA7,LAMA5,LAMC1,PPP1R12A,PDGFA,PDGFRB,MAPK3,PTEN,ROCK1,THBS2,ITGA10,ROCK2,ITGA11,PARVA,PDGFC,TLN2,SHC4,FGF13,MYH10,WASL,ARPC1A,ARHGEF12,GNG12,PIP4K2C
hsa04270	Vascular smooth muscle contraction	16/120	−3.8133	ADCY3,AGTR1,CALD1,EDNRA,GNA11,KCNMB1,MYH11,MYL6,PPP1R12A,MAPK3,PTGIR,ROCK1,ROCK2,RAMP1,ARHGEF12,PPP1R14A
hsa00071	Fatty acid degradation	9/44	−3.7701	ACADL,ACAT1,ACAT2,ADH1A,ADH1B,ALDH2,ALDH3A2,ECI1,HADH,ACYP2,LDHA,LDHB,ACSS2,PFKM,PGM1
hsa03020	RNA polymerase	7/31	−3.3221	POLR2C,POLR2F,POLR2G,POLR2H,POLR2I,POLR3F,POLR3C,ADCY3,AK1,GUK1,NME3,PGM1,PRPS2,ENPP4,NME7,AK3,NUDT9,POLE4,NT5C3B,CTPS1
hsa05016	Huntington’s disease	20/193	−3.1152	COX5B,COX6C,COX7A1,COX7B,COX7C,HDAC2,NDUFA4,NDUFA8,NDUFB10,NDUFC1,POLR2C,POLR2F,POLR2G,POLR2H,POLR2I,SOD1,ATP5H,UQCRQ,NDUFA12,NDUFA4L2,UBE2G2,SNCAIP,PINK1,COX17,ATP6V1D,CAPN2,MAPK3,RYR3
hsa05200	Pathways in cancer	33/397	−2.9576	ADCY3,AGTR1,AR,COL4A1,E2F3,EDNRA,MECOM,FGF13,FOXO1,FZD2,GNA11,GSTP1,MSH6,HDAC2,HRAS,LAMA5,LAMC1,SMAD4,PDGFA,PDGFRB,MAPK3,PTEN,ROCK1,SLC2A1,TCEB1,ZBTB16,FZD3,CCDC6,ROCK2,GNB5,RALBP1,ARHGEF12,GNG12,PDGFC
hsa05412	Arrhythmogenic right ventricular cardiomyopathy (ARVC)	10/74	−2.6366	ACTN1,CACNB3,CDH2,DAG1,GJA1,ITGA7,RYR2,SGCA,ITGA10,ITGA11
hsa00520	Amino sugar and nucleotide sugar metabolism	7/48	−2.1749	CYB5R3,GMDS,PGM1,PMM1,UAP1,UGDH,UGP2

List in term: the number of DEGs on the total number of genes in GO term

*Log p* logarithm processing of *p* value

### Establishment of TFs-target genes regulatory network

**Fig. 7:**
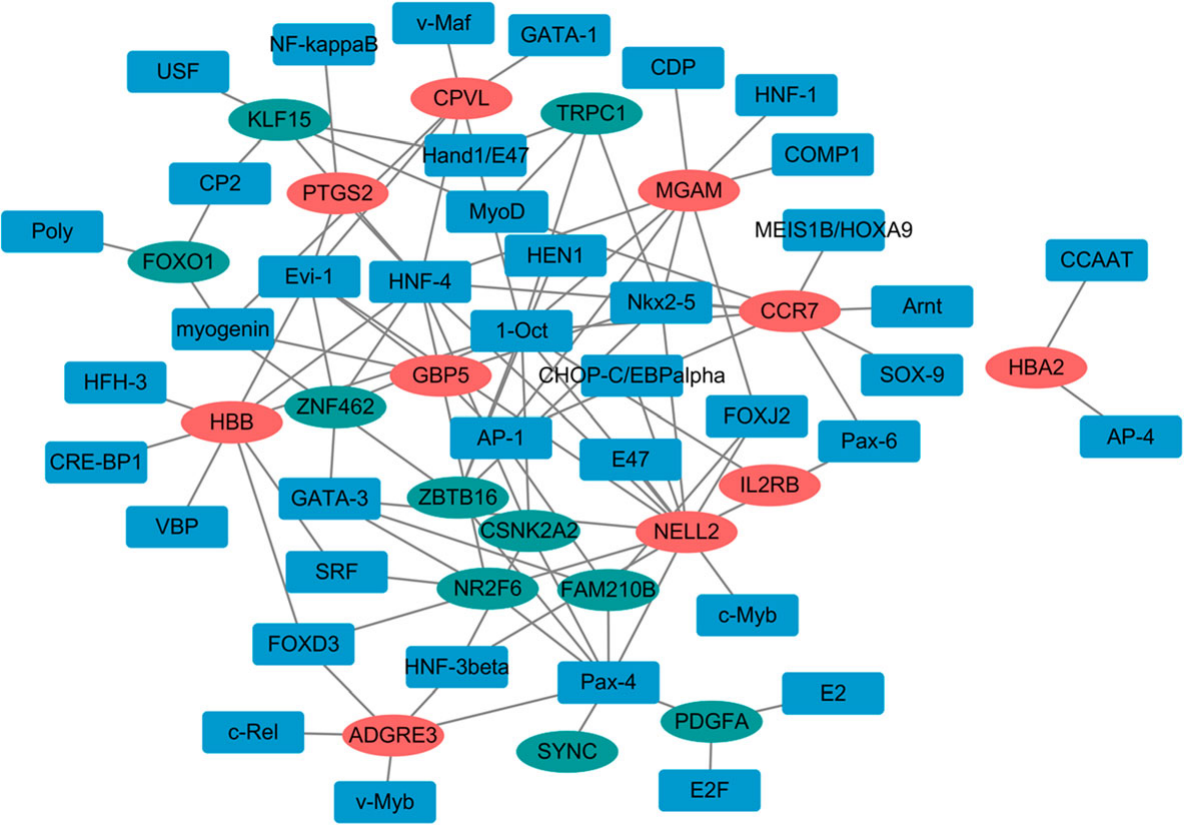
The TFs networks of the top 20 DEGs. Diamonds and ellipses represent the TFs and target genes, respectively. The red and green colors represent up-regulation and down-regulation, respectively

In order to study the TFs-target genes regulatory network for AAA, we utilized TRANSFAC to identify TFs regulating the top ten up-regulated or down-regulated DEGs. In the end, we obtained transcriptional regulatory networks comprised of 190 pairs of TFs-genes involving 40 TFs (Fig. 7). In this network, the top 7 downstream genes covered by most TFs were neural EGFL like 2 (NELL2, degree = 13), C-C motif chemokine receptor 7 (CCR7, degree = 9), maltase-glucoamylase (MGAM, degree = 8), hemoglobin subunit beta (HBB, degree = 8). Five hub TFs were HNF-4 (degree = 10), Oct-1 (degree = 10), Pax-4 (degree = 8), Evi-1 (degree = 6) and Nkx2–5 (degree = 6) (Table 5).

**Table 5:** Top 5 TFs and target genes

TFs	Number	Target genes
Oct-1	10	CCR7, CPVL, CSNK2A2, HBB, IL2RB, MGAM, NELL2, TRPC1, ZBTB16, ZNF462
HNF-4	10	CCR7, CPVL, CSNK2A2, HBB, KLF15, MGAM, NELL2, NR2F6, PTGS2, ZNF462
Pax-4	8	ADGRE3, CSNK2A2, FAM210B, NELL2, NR2F6, PDGFA, SYNC, ZBTB16
Evi-1	6	CPVL, GBP5, HBB, NELL2, PTGS2, ZNF462
Nkx2–5	6	CCR7, GBP5, MGAM, NELL2, TRPC1, ZBTB16

### PPI network

**Fig. 8:**
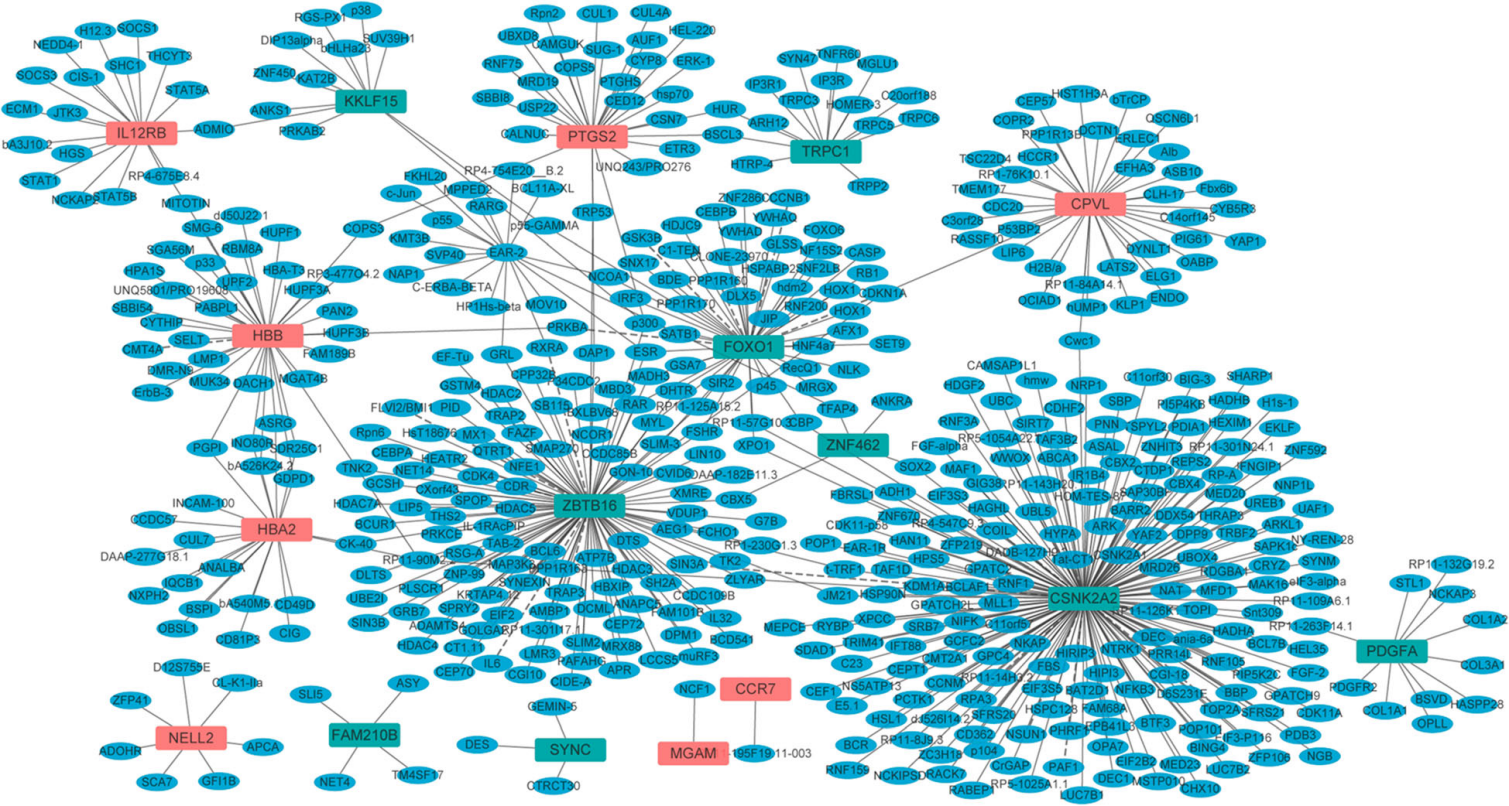
The PPI networks of the top 20 DEGs. All the diamonds are proteins encoded by the top 20 DEGs and the blue ellipses represent other proteins. The red and green colors represent up-regulation and down-regulation, respectively

To obtain the interaction between the proteins encoded by DEGs and other proteins, PPI network was explored and visualize by Cytoscape. PPI networks of the top 10 up-regulated and the top 10 down-regulated DEGs were presented in Fig. 8. As Fig. 8 shows, the network consisted of 539 nodes and 567 edges. The red and green diamonds indicate the up- and down-regulated genes in AAA, respectively. The blue ellipses present the proteins that interacted with those proteins encoded by DEGs. The top three proteins with a high degree were casein kinase 2 alpha 2 (CSNK2A2, degree = 184), zinc finger and BTB domain containing 16 (ZBTB16, degree = 113) and forkhead box O1 (FOXO1, degree = 53).

### Validation of CCR7 and PDGFA in AAA

**Fig. 9:**
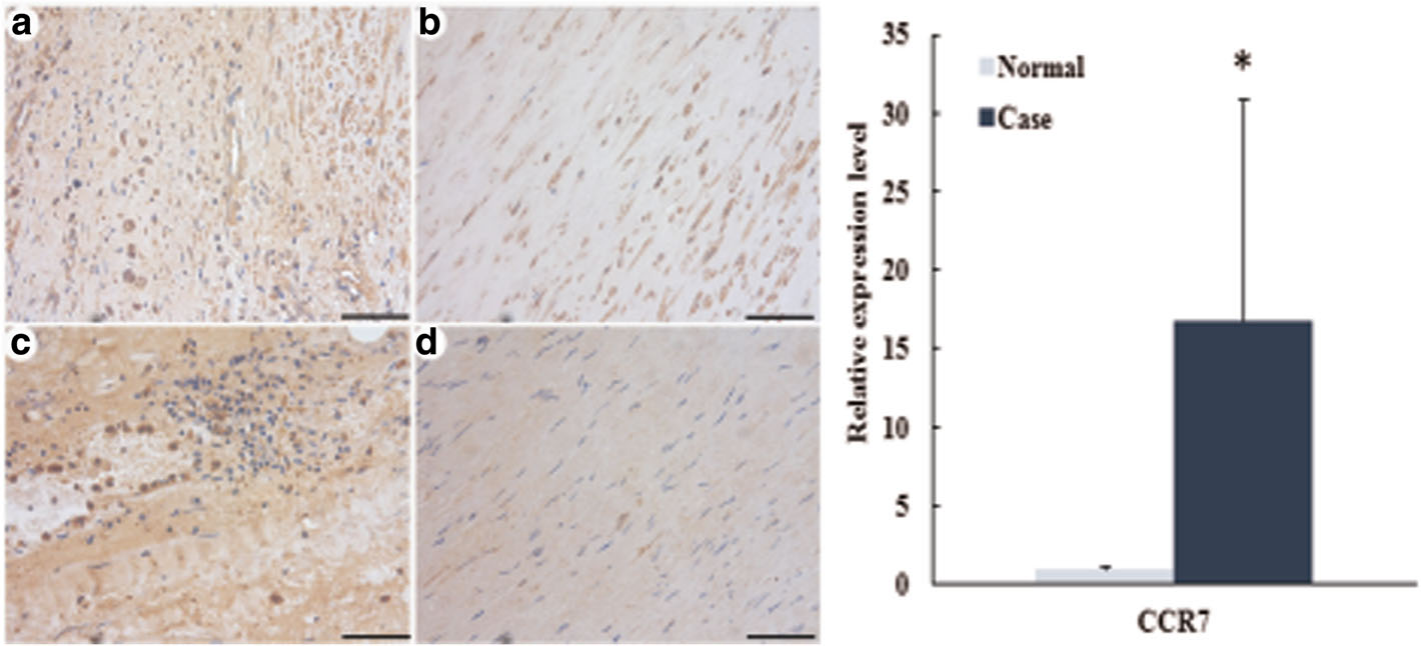
The IHC staining of CCR7 in AAA. CCR7 protein expression level detected by immunohistochemistry and photographs was amplified 10 × 20 multiples. Bar = 100 μm. **a** and **c** were the case samples from two patients with AAA; **b** and **d** were the control sample from two normal individuals. ^*^P<0.05 vs control

**Fig. 10:**
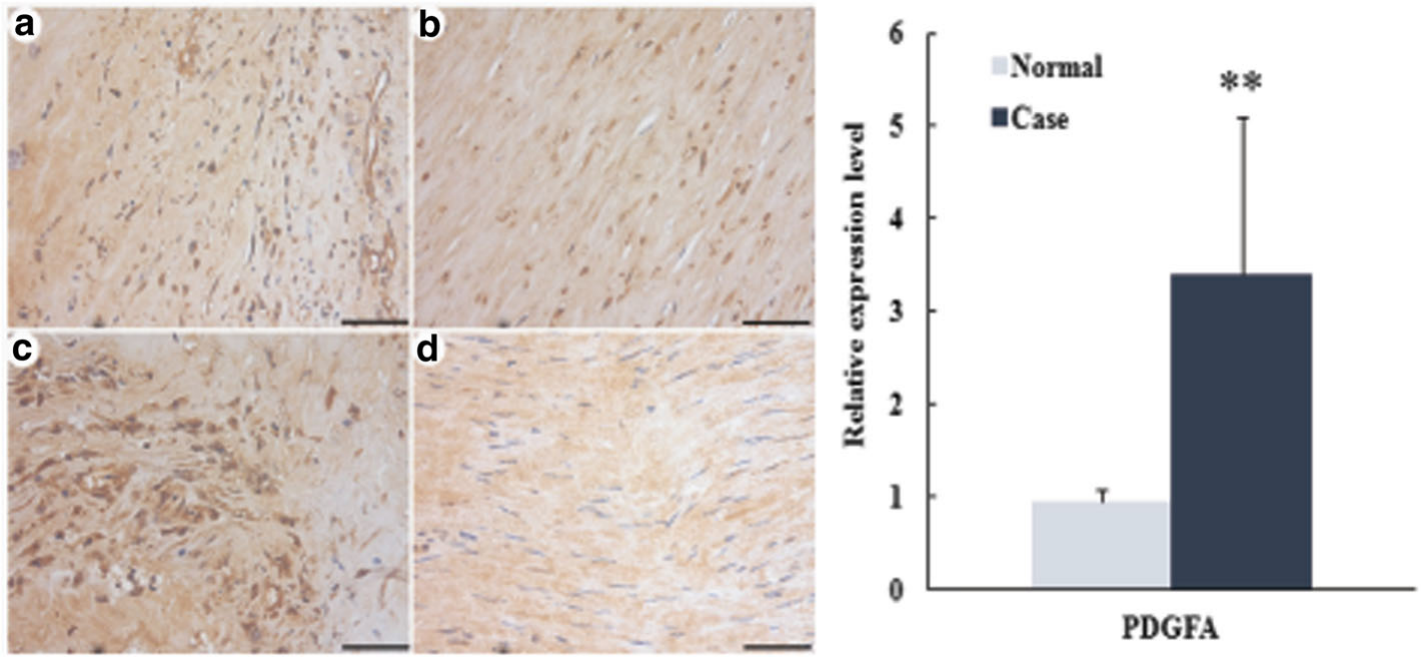
The IHC staining of PDGFA in AAA. PDGFA protein expression level detected by immunohistochemistry and photographs was amplified 10 × 20 multiples. Bar = 100 μm. **a** and **c** were the case samples from two patients with AAA; **b** and **d** were the control sample from two normal individuals. ^**^P<0.01 vs control

In order to validate the expression of CCR7 and platelet derived growth factor subunit A (PDGFA), we assessed the protein expression of CCR7 and PDGFA in AAA through the immunohistochemistry (Fig. 9 and Fig. 10). The result showed that CCR7 was obviously up-regulated in AAA compared with the control, which was consistent with the bioinformatic consequence. However, PDGFA was significantly up-regulated in AAA compared with the control, which was not in line with the bioinformatic result.

### ROC curve analysis

**Fig. 11:**
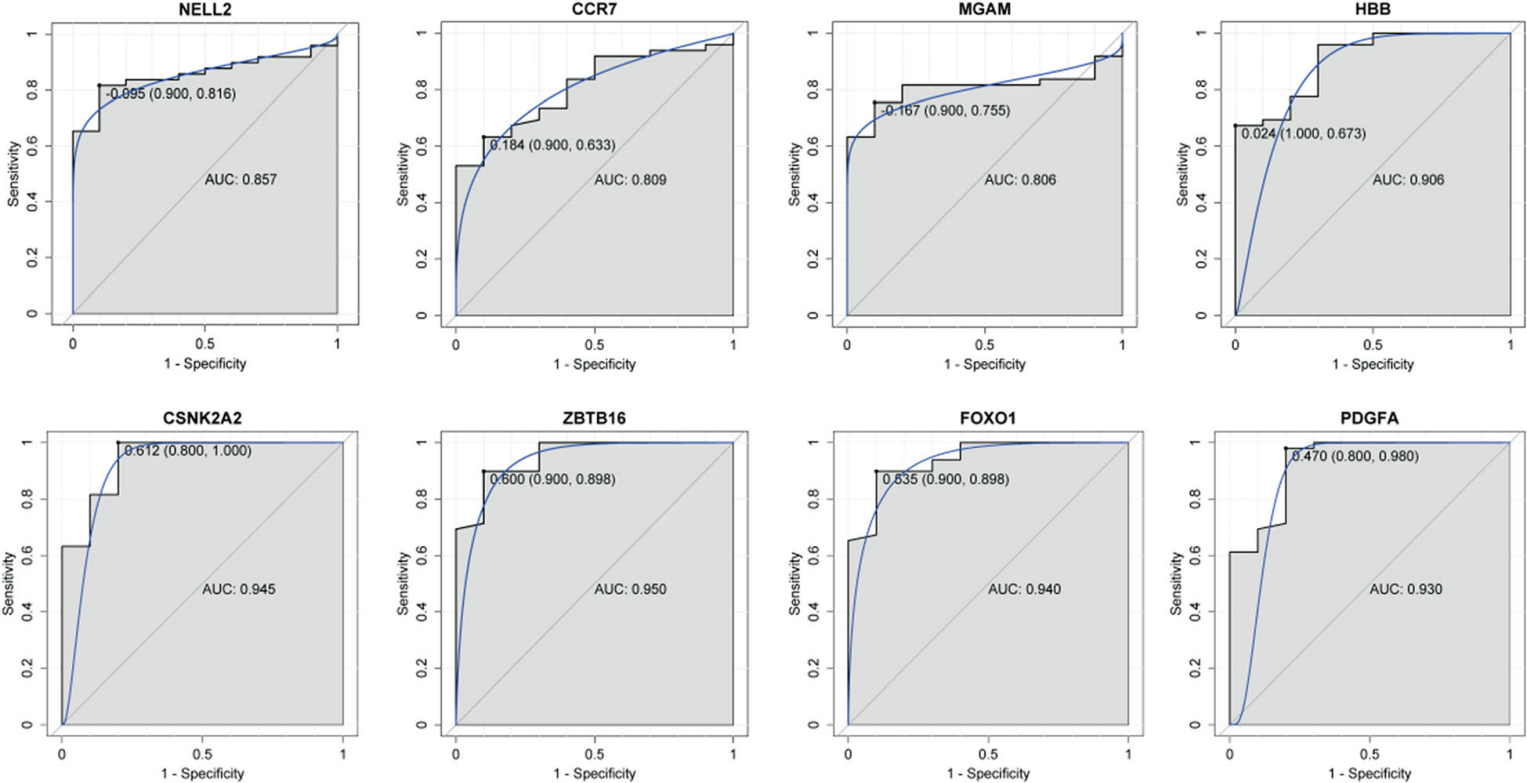
The ROC curve analyses of NELL2, CCR7, MGAM, HBB, CSNK2A2, ZBTB16, FOXO1 and PDGFA between AAA and healthy controls

In order to access the discriminatory ability of the NELL2, CCR7, MGAM, HBB, CSNK2A2, ZBTB16, FOXO1 and PDGFA among AAA tissues and adjacent non-tumor tissues generated from GEO database, ROC curve analyses were conducted and AUC were calculated. As Fig. 11 shown, the AUC of all these genes was more than 0.8. For AAA diagnosis, the sensitivity and specificity of these genes were very high.

## Discussion

In spite of improvement to surgical techniques that have been made in AAA treatment, morbidity and mortality after operations are still common. AAA seriously influences the life quality of patients and brings a heavy burden on the family. Therefore, it is urgent to elucidate AAA pathogenesis mechanism for developing novel diagnose and therapeutic target.

TFs are key regulatory factors in gene expression. The construction of regulatory networks between TFs and target genes is helpful in understanding the biological regulatory mechanism in the development of AAA. In this study, we found NELL2, CCR7, MGAM and HBB were significantly expressed genes with the most degree under the regulation of TFs including HNF-4, Oct-1 and Pax-4. NELL2 is a neural tissue-enriched protein in mammal and it is a receptor for vascular endothelial growth factor-A, which plays an important role in angiogenesis. It is reported that the mRNA expression of NELL2 is up-regulated in benign prostate hyperplasia and prostate cancer [22]. In addition, NELL2 is regarded as the potential biomarker for bladder cancer [23]. CCR7 is a pro-inflammatory cytokine and is found in human atherosclerotic plaques [24]. It is found that expression of CCR7 is dramatically down-regulated in human carotid atherosclerotic plaques [25]. MGAM is found down-regulated and considered as a candidate serum biomarker in colorectal cancer [26]. Additionally, MGAM is a significantly mutated gene in lung adenocarcinoma [27]. HBB is suggested as a potential biomarker in the plasma sample of patients with AAA [28]. In this study, we found that NELL2, CCR7, MGAM and HBB were up-regulated in the AAA tissues, which played crucial roles in the carcinogenesis of AAA.

The interaction among proteins determines the characteristic of the cell, tissue and individual. The study of PPI is a useful way to find the potential drug target of AAA. Herein, we found three genes including CSNK2A2, ZBTB16 and FOXO1 were for the most degree in the PPI network. CSNK2A2 is found to be correlated with ovarian cancer patient survival. Furthermore, the down-regulation of CSNK2A2 will decrease the proliferation of ovarian cancer cells [29]. ZBTB16, also called PLZF, plays an important role in oncogenesis and is first identified in acute promyelocytic leukemia [30]. Based on a microarray study, ZBTB16 is found to be down-regulated in AAA [31]. FOXO1 is a transcription factor and plays roles in diverse physiological processes including Akt-dependent cell proliferation and apoptosis [32]. Additionally, FOXO1 is also involved in energy metabolism and autophagy [33]. In our study, we found down-regulated expression of CSNK2A2, ZBTB16 and FOXO1. It is worth mentioning that ZBTB16 and FOXO1 were also involved in the pathways in cancer according to the KEGG analysis. In addition, PDGFA was one of the top ten down-regulated genes and also involved in the pathways in cancer. PDGFA is expressed in vascular smooth muscle cells and has been involved in migration and proliferation of vascular smooth muscle cells [34]. Moreover, the importance of PDGFA in the arterial system has been demonstrated on account of that fact that the proliferation of arterial vascular smooth muscle cells was strongly stimulated by PDGFA [35]. Moreover, an in situ hybridization study has demonstrated mRNA for PDGFA in atherosclerotic plaques [36]. In this study, we found that PDGFA was down-regulated in AAA. However, the IHC result was not consistent with the bioinformatic analysis. The small sample size may account for the discrepancy. In a word, CSNK2A2, ZBTB16 and FOXO1 played a crucial role in the oncogenesis of AAA and could be considered as drug targets of AAA.

Apart from the cancer signal pathway, vascular smooth muscle contraction was another signal pathway identified that associated with AAA. Vascular smooth muscle cells have been shown to play an important synthetic role in vascular remodeling [37, 38]. It is pointed out that vascular smooth muscle cells are the main component of the aortic media and the dysfunction plays a key role in different arterial diseases, such as AAA [39]. In addition, vascular smooth muscle cell activation is the main hallmark of atherosclerosis, which is a risk factor of AAA [40–43]. Several down-regulated genes were significantly involved in the signal pathway, such as AGTR1, CALD1, EDNRA, MYH11, RAMP1, ROCK1 and ROCK2.

Angiotensin II receptor type 1 (AGTR1) is a cardiovascular risk gene. Jones, G. T et al. found that AGTR1 was remarkably associated with AAA [44]. In addition, the 1166A *>* C polymorphism in AGTR1 has been demonstrated to be associated with AAA [45, 46]. It is noted that AGTR1 blockers (ARBs) have been investigated for prevention or delay of aortic dilatation [47]. It is reported that the expression of caldesmon 1 (CALD1) is increased in aortas, which protects from aneurysm. This suggested that importance of CALD1 in maintaining vascular integrity in AAA. Endothelin receptor type A (EDNRA) is primarily located in the vascular smooth muscle cells and mediates vasoconstriction and proliferation [48]. It has been reported that EDNRA on chromosome 4q31 is related to intracranial aneurysm [49]. It is found that heterozygous mutation of myosin heavy chain 11 (MYH11) results in the early and severe decrease in the aortic wall elasticity [50]. Additionally, it has been demonstrated the relationship between MYH11 genetic and epigenetic and thoracic aortic aneurysms and dissections [51, 52]. Receptor activity modifying protein 1 (RAMP1) is a member of a family of calcitonin receptor modifying proteins and is thought to play an important role in regulating blood pressure by vascular relaxation. Tsujikawa K et al. found that ramp1-deficient mice exhibited elevated blood pressure [53]. It is pointed out that the mRNA levels of RAMP1 are decreased in AAA [31]. It is found that the expression of Rho associated coiled-coil containing protein kinase 1 (ROCK1) and Rho associated coiled-coil containing protein kinase 1 (ROCK2) was increased at the AAA lesion compared with control [54]. Thus it can be seen that AGTR1, CALD1, EDNRA, MYH11, RAMP1, ROCK1 and ROCK2 played an important role in vascular smooth muscle contraction, which was significantly associated with AAA.

In order to access the discriminatory ability of identified genes in the TFs and PPI network, eight genes including NELL2, CCR7, MGAM, HBB, CSNK2A2, ZBTB16, FOXO1 and PDGFA were applied to ROC curve analyses among AAA tissues and adjacent non-tumor tissues in GEO database. Our result showed that the AUC of all these genes was more than 0.8, especially HBB (AUC: 0.906), CSNK2A2 (AUC: 0.945), ZBTB16 (AUC: 0.950), FOXO1 (AUC: 0.940) and PDGFA (AUC: 0.930). This suggested that NELL2, CCR7, MGAM, HBB, CSNK2A2, ZBTB16, FOXO1 and PDGFA may have value in diagnosis of the development of AAA.

## Conclusions

In summary, we found a series of DEGs in AAA. Among which, eight genes including NELL2, CCR7, MGAM, HBB, CSNK2A2, ZBTB16, FOXO1 and PDGFA could be used for the diagnosis biomarkers of AAA. Especially, CSNK2A2, ZBTB16 and FOXO1 could be considered as drug targets in the therapy of AAA. In addition, vascular smooth muscle contraction was an important signal pathway identified in this study, which played crucial roles in the aortic angiogenesis of AAA. There are limitations to our study. Firstly, the sample size in the IHC experiment is small and large numbers of tissue samples are needed to further validate the identified DEGs. Secondly, biological function of identified DEGs is not investigated, some in vivo or in vitro experiments are needed to further study the molecular mechanism of AAA. Thirdly, the sample size of normal individuals in the selected dataset is less than that of the patient group. In further studies, it is better to sample equal numbers of individuals in both groups in order to reduce the false positive/negative rate for up−/down-regulated DEGs detection.

## Abbreviations

AAA: abdominal aortic aneurysm
AGTR1: angiotensin II receptor type 1
ARBs: AGTR1 blockers
AUC: area under the curve
CALD1: caldesmon 1
CCR7: C-C motif chemokine receptor 7
CSNK2A2: casein kinase 2 alpha 2
DAB: diaminobenzidine
DEGs: differentially expressed genes
EDNRA: endothelin receptor type A
FDR: false discovery rate
FOXO1: forkhead box O1
GEO: gene expression omnibus
HBB: hemoglobin subunit beta
IHC: immunohistochemical
MGAM: maltase-glucoamylase
MYH11: myosin heavy chain 11
NELL2: neural EGFL like 2
PDGFA: platelet derived growth factor subunit A
PPI: protein-protein interaction
RAMP1: receptor activity modifying protein 1
ROC: receiver operating characteristic
ROCK1: Rho associated coiled-coil containing protein kinase 1
ROCK2: Rho associated coiled-coil containing protein kinase 1
TFs: transcriptional factors
ZBTB16: zinc finger and BTB domain containing 16
